# Raltegravir, elvitegravir, and metoogravir: the birth of "me-too" HIV-1 integrase inhibitors

**DOI:** 10.1186/1742-4690-6-25

**Published:** 2009-03-05

**Authors:** Erik Serrao, Srinivas Odde, Kavya Ramkumar, Nouri Neamati

**Affiliations:** 1 Department of Pharmacology and Pharmaceutical Sciences, University of Southern California, School of Pharmacy, 1985 Zonal Avenue, Los Angeles, CA 90089, USA

## Abstract

Merck's MK-0518, known as raltegravir, has recently become the first FDA-approved HIV-1 integrase (IN) inhibitor and has since risen to blockbuster drug status. Much research has in turn been conducted over the last few years aimed at recreating but optimizing the compound's interactions with the protein. Resulting me-too drugs have shown favorable pharmacokinetic properties and appear drug-like but, as expected, most have a highly similar interaction with IN to that of raltegravir. We propose that, based upon conclusions drawn from our docking studies illustrated herein, most of these me-too MK-0518 analogues may experience a low success rate against raltegravir-resistant HIV strains. As HIV has a very high mutational competence, the development of drugs with new mechanisms of inhibitory action and/or new active substituents may be a more successful route to take in the development of second- and third-generation IN inhibitors.

## Overview

Though many potent inhibitors of the viral life cycle have arisen over recent years, HIV persists as a global pandemic with eradication unlikely in the near future. Over 33 million people, including 2.5 million children, are living with HIV worldwide as of December, 2007 [1]. Almost 7000 people are newly infected with HIV, and around 6000 die from AIDS, each day. Due to the lack of education about risky behaviors and the lack of access to treatment, low- and middle-income countries remain the largest producers of new HIV infections, with AIDS being the leading cause of death in Sub-Saharan Africa. Five percent of all adults are living with HIV or AIDS in this region [1,2]. Worldwide spending on HIV/AIDS research, treatment, and prevention has risen from $300 million in 1996 to an estimated $10 billion in 2007, but the global need is projected to be much higher [2,3]. Although novel estimation procedures have contributed to a more accurate, reduced 2008 global estimate of those living with HIV and AIDS in comparison to the past few years, this number remains staggering and ever increasing [1,4].

The advent of highly active antiretroviral therapy (HAART) has brought with it a significant decrease in AIDS-related deaths over the last ten years. Prior to the development of raltegravir, HAART had been recommended to consist of at least three different drugs targeting separate stages of the HIV life cycle: two nucleoside reverse transcriptase inhibitors, plus either a non-nucleoside reverse transcriptase inhibitor such as efavirenz, or a protease inhibitor [5,6]. Studies have shown that effective administration of these HAART regimens can result in a large-scale decrease in plasma levels of viral RNA, as well as a significant increase in CD4 cell count [7-9]. Furthermore, HAART has been shown to reduce the incidence of opportunistic infections and HIV-associated cancers, contributing to the significantly decreased number of HIV- and AIDS-related deaths each year (and correspondingly contributing to the much increased amount of people living with the disease each year) [10]. However, HAART regimens have been incapable of viral eradication, due in part to the viral establishment of reservoirs within latently infected and resting CD4^+ ^T cells and CD8^+ ^T cells [11-13]. Also, HAART has frequently led to the emergence of drug resistant viral strains [14,15]. Hence, much innovation is essential for the success of future anti-HIV drug research.

An area of much recent progress has been that of HIV-1 IN inhibitor design. IN is an essential enzyme for viral replication, and it has no human homolog [for a recent review, see Reference [16]]. IN catalyzes the insertion of reverse transcribed viral cDNA into the host cell genome via a multi-step process. The first step in integration occurs in the host cell cytosol and is referred to as 3'-processing. During this step, IN cleaves a dinucleotide from each viral DNA terminus at a conserved CA sequence, yielding two reactive 3' hydroxyl groups. Following this processing step, IN associates with a number of viral and cellular proteins, forming a pre-integration complex (PIC), and then migrates to the nucleus. Within the nucleus the reactive hydroxyl groups are utilized in nucleophilic attack upon the host cell genome, a process known as strand transfer [17]. IN multimerization is also required for formation of the PIC. As a dimeric IN species is required for 3'-processing, the strand transfer step calls for a tetrameric IN arrangement. Proper integration of viral DNA into the host cell genome leads to viral protein expression, maturation, and propagation [18]. IN catalysis is vital to proper HIV-1 replication and sustained infection, and potent small-molecule IN inhibitors have been avidly sought over the last ten years as a supplement to HAART and a novel angle of attack against drug resistant viruses.

## The birth of the diketo acids and the emergence of raltegravir

A previous large-scale, random screen of over 250,000 compounds yielded potent inhibitors, and the most active compounds proved to be 4-aryl-2,4-diketobutanoic acids, containing a distinct β-diketo acid (DKA) moiety that was capable of coordinating metal ions within the IN active site [19]. The active DKA containing compounds from this study showed a significant preference for strand transfer inhibition over that of 3'-processing *in vitro*. For example, the most potent compound, L-731,988, exhibited a 70-fold higher IC_50 _value of 6 μM for 3'-processing compared to its 80 nM IC_50 _value for strand transfer inhibition. Importantly, L-731,988 exerted a completely inhibitory effect upon HIV-1 infection in a cell-based assay at a concentration of 10 μM. In a follow-up study [20], it was found that the DKA and target DNA binding sites on IN overlap and are both distinct from that of the viral DNA, and also that the DKAs bind with a 1000-fold higher affinity to IN in complex with 3'-processed viral DNA than to non-complexed IN (10–20 μM versus 100 nM).

**Figure 1:**
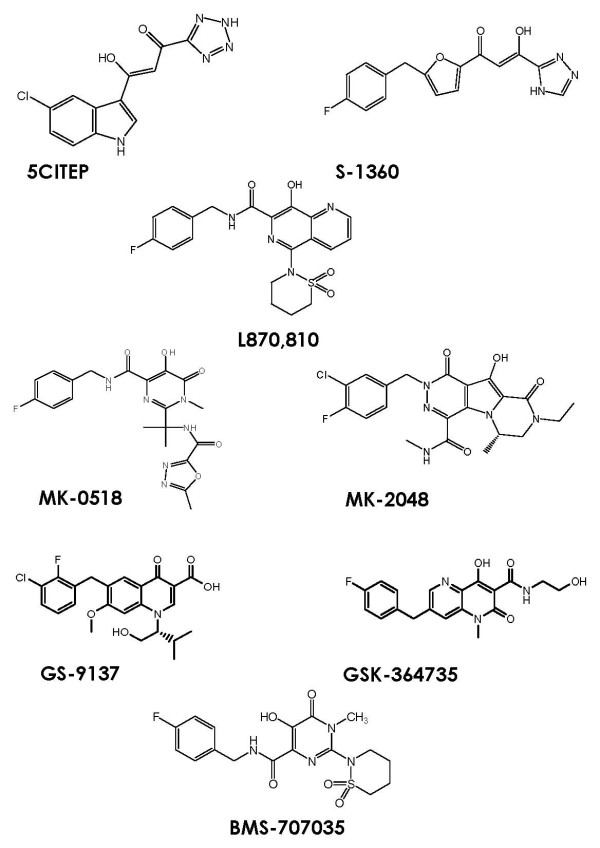
**The structure of diketo acid-based HIV-1 integrase inhibitors**.

Simultaneously, a different group discovered and developed potent DKA compounds, leading to both the first inhibitor co-crystallized with IN (5CITEP, Figure 1) and the first clinically tested inhibitor (S-1360, Figure 1). 5CITEP was included in this group's 1999 patent [21], which covered DKAs containing various indole and substituted indole groups. Specifically, 5CITEP possessed a tetrazole group in place of the common DKA carboxylic acid moiety. 5CITEP inhibited IN 3'-processing and strand transfer at IC_50 _values of 35 μM and 0.65 μM, respectively [22], and it was subsequently reported in complex with IN in the vicinity of the active site residues Asp-64, Asp-116, and Glu-152, providing the first crystal structure information about IN [23]. Further modification led to the inclusion of heterocyclic groups in place of the indoles, culminating in the development of multiple nitrogen and oxygen-containing heterocyclic analogs, all of which were covered in a 2000 patent [24]. S-1360, or (*Z*)-1-[5-(4-fluorobenzyl)furan-2-yl]-3-hydroxy-3-(1*H*-1,2,4-triazol-3-yl)propenone, was the most promising of these compounds and went on to become the first clinically tested HIV-1 IN inhibitor. It exhibited a 20 nM IC_50 _for IN inhibition *in vitro*, and it accomplished inhibition of HIV replication in MTT assays with EC_50 _and CC_50 _values of 200 nM and 12 μM, respectively [25,26]. Acceptable safety and toxicology profiles were attained in animal models, and Phase I trials showed good pharmacokinetics in a group of 24 healthy HIV-negative humans [25]. However, S-1360 failed efficacy studies due to its reduction in humans at the carbon linked to the triazole heterocycle, yielding an inactive metabolite that was rapidly cleared through glucuronidation in the non-cytochrome P450 pathway [27], and its development was soon abandoned.

The DKA pharmacophore was subsequently transferred to a naphthyridine carboxamide core, conferring similar antiviral activity and strand transfer selectivity [28]. The most active inhibitor from this class, L870,810 (Figure 1), showed very promising activity, with IC_50 _values as low as 4 nM against multidrug-resistant viruses [29]. L870,810 soon became the second IN inhibitor to enter clinical trials. However, liver and kidney toxicity surfaced after long-term treatment in dogs, bringing a premature end to the drug's clinical progress [30]. This relative success with diketo acid structural analogs led to the derivation of a class of *N*-alkyl hydroxypyrimidinone carboxylic acids, which showed nanomolar activity against HIV-1 IN in enzymatic assays and a good pharmacokinetic profile (modest oral bioavailability, low plasma clearance, and good half-life) in rats [31]. MK-0518, also known as raltegravir (Figure 1), emerged as the most promising pyrimidinone carboxamide derivative and soon became the first IN inhibitor to progress into Phase III clinical trials. Though multiple resistant mutations have surfaced in both treatment-experienced and treatment-naïve patients [32], MK-0518 has exhibited low nanomolar and strand transfer selective *in vitro *IN inhibition, an IC_95 _value of 31 nM in the presence of normal human serum (NHS), and synergistic effects in combination with multiple current antiretroviral drugs [15,33]. Raltegravir (a.k.a. Isentress™) became the first FDA approved IN inhibitor in October of 2007 and is currently being administered as a new addition to HAART regimens.

## Me-too drugs

Comparable to every innovation, promising new drugs will be quickly followed into the market by multiple analogs, most striking in their similarity to the original. With an average cost of $2 billion to bring a single drug to market [34] and only one in three drugs producing revenues that match or exceed these average research and development costs [35], one can imagine the temptation for pharmaceutical companies to forego the pains of innovation and rather simply modify current leads. There have been differences of opinion regarding the value of these so-called "me-too" drugs [36,37]. Some view that me-too products are essential for drug optimization and progress, and that they generate vital marketplace competition, leading to better quality and lower costs. Still others argue that slight structural modifications producing negligible improvements in drug activity are a waste of time and effort, and that the vast amount of money spent on competitive advertisement could be invested instead into actual innovation or the development of orphan drugs. One of the clearest examples of me-too product generation can be seen in the statin drug market. There are currently six 3-hydroxymethylglutaryl coenzyme A reductase inhibitors (statins) commercially available. However, there has yet to be a large, randomized trial comparing the clinical effects of equivalent doses of each statin upon prevention of vascular disease. The six drugs differ slightly in pharmacokinetics, and knowledge gained throughout their design and development about the health implications of high cholesterol has been beneficial. However, their structures, functions, and clinical effects are highly homologous, and over 90% of physicians have been shown to utilize at most three different statins for all of their incident prescribing [38]. Another obvious instance of me-too production has been the evolution of AstraZeneca's Prilosec (omeprazole) to Nexium (esomeprazole). There are only two differences between the two drugs – Prilosec contains a racemic mixture of the D- and S-isomers of omeprazole while Nexium contains solely the more potent S-isomer, and Nexium is protected by patent and far more expensive than Prilosec. Furthermore, Nexium has been shown in clinical trials to be only marginally more effective than Prilosec in control of stomach acid levels [39]. Though there have been several examples of me-too drugs providing a substantial increase in efficaciousness or decrease in toxicity – such as derivatives of the anthracycline chemotherapeutic daunorubicin [40] and the beta blocker propanolol [41] – very few FDA approved me-too drugs actually exhibit a significant enhancement of activity in comparison to their predecessors. In fact, of the 1035 drugs approved by the FDA between 1989 and 2000, only 361 contained new active substituents, and less than half of these received a priority FDA review due to the low likelihood of providing a significant advantage over existing treatments [42].

An area in which me-too drug generation has been especially prevalent recently is that of HIV-1 IN inhibitor design. Although raltegravir has become a modern blockbuster anti-HIV drug, multiple viral amino acid mutations have already been identified that confer robust viral resistance to the drug [43]. Specifically, mutations causing invulnerability to raltegravir have been shown to contribute to an almost 25% virological failure rate within 48 months of treatment [44]. This viral drug resistance most often results from the substitution of one of three amino acids – Y143, Q148, or N155 – usually in combination with at least one other mutation [44]. The specific substitutions of G140S and E92Q are typically associated with N155 and Q148 mutations, and the G140S/Q148H/R double substitution has been shown to result in a >400-fold viral resistance to raltegravir [45]. While the G140S mutation displays only a weak resistance to raltegravir (IC_50 _= 30 nM), the Q148H IN mutant is strongly resistant (IC_50 _> 700 nM). Interestingly though, G140S has recently been shown to effectively restore the poor replication ability of Q148H to near WT levels, illustrating its compensatory nature [46]. Even with this resistance profile, raltegravir has been the target of an excessive amount of me-too research and development over the last two years. Though, again, there have been historical instances of me-too drugs significantly benefiting patients and instigating medical progress, they have for the most part only benefited pharmaceutical companies. Although it is definitely possible that the next blockbuster anti-HIV drug could be a raltegravir lookalike, we hypothesize that raltegravir me-too drugs, targeting a virus that exhibits an extraordinary rate of resistance evolution, will experience a low probability of success in the clinical setting due to viral resistance and cross-resistance issues.

## Me-too or second generation?

**Figure 2:**
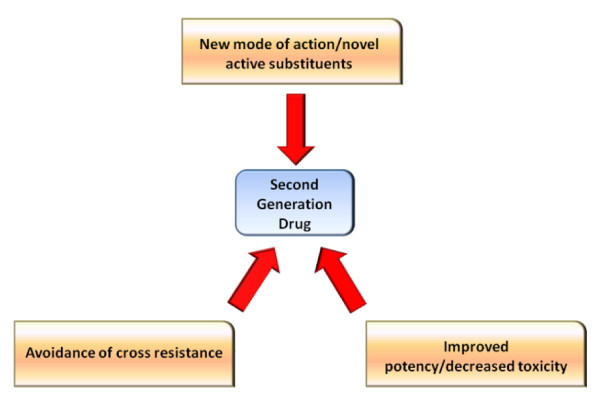
**Requirements for "second generation drug" classification**.

In contrast to me-too drugs, second generation HIV-1 IN inhibitors benefit patients. In order to be considered a bona fide second generation inhibitor, a compound of interest must meet at least one of three criteria (Figure 2). First, a second generation inhibitor may exhibit a new mode of action and/or contain novel active substituent(s). A second generation inhibitor may also possess significantly improved potency and/or significantly decreased toxicity. Thirdly, a second generation inhibitor may exhibit potency while avoiding cross-resistance from mutants resistant to similar drugs. Obviously, the more criteria a selected drug meets, the more success it will enjoy in the clinical setting and in the global market. A recent example of a second generation drug that has narrowly avoided me-too labeling is the protease inhibitor, darunavir. Darunavir is the 10^th ^protease inhibitor to be marketed in the United States, and it was approved by the FDA on June 23, 2006. Darunavir's chemical structure is almost identical to its precursor, amprenavir, in that it simply contains a double-ringed terminal *bis*-tetrahydrofuran group in place of the single-ringed terminal tetrahydrofuran on amprenavir. Additionally, darunavir and amprenavir occupy a highly overlapping volume in the protease active site. However, darunavir's two additional oxygen atoms upon its *bis*-tetrahydrofuran moiety contribute to a two order of magnitude increase in binding affinity in comparison to amprenavir, by forming strong hydrogen bonds with the main chain atoms of amino acids Asp-29 and Asp-30 [47]. This tighter binding leads to an increased ability of darunavir to fit within the protease envelope and to exhibit potent activity against even multi-drug resistant viral strains. Darunavir specifically retains nanomolar IC_50 _values in the presence of mutations resistant to ritonavir, nelfinavir, indinavir, saquinavir, and even amprenavir (mutations at L10F, V32I, M46I, I54M, A71V, and I84V) [48]. So, although darunavir's structural and mechanistic properties are me-too-like, its resistance profile created by its relatively high binding affinity is much different than all preexisting protease inhibitors. It is therefore considered a second generation drug. The structural and mechanistic properties of recent raltegravir me-too compounds are highly analogous, as are the pharmacokinetics. We predict that the resistance profiles will be nearly identical as well, precluding much clinical success.

## Raltegravir me-too analogs

Most of the recent raltegravir me-too drugs comply with the general diketo acid pharmacophore structural requirements – or a hydrophobic aromatic (usually fluorobenzyl) component and a variable acidic component linked to either side of a DKA linker (Figure 1). This linker usually consists of a γ-ketone, an enolizable α-ketone, and a carboxylic acid, but the carboxylic acid has been substituted with other acidic (tetrazole and triazole) and basic (pyridine) bioisosters [49]. Whereas the aromatic DKA pharmacophore substituent confers strand transfer selectivity, the acidic component contributes to 3'-processing inhibitory potency [50,22].

## Clinically tested me-too IN drugs

### MK-2048

Research into second generation DKA inhibitors shortly after the FDA approval of MK-0518 led to the design of a set of tricyclic hydroxypyrroles that mimicked the common DKA metal binding pharmacophore. Optimization of a derived set of 10-hydroxy-7,8-dihydropyrazinopyrrolopyrazine-1,9-dione compounds resulted in one of the first raltegravir me-too leads, MK-2048 (Figure 1). MK-2048 has exhibited an IC_95 _of 40 nM in the presence of 50% NHS, favorable pharmacokinetics, and potent antiretroviral activity against four IN mutants displaying raltegravir resistance [51,52].

### GS-9137 (elvitegravir)

Early modification of the DKA motif by Japan Tobacco resulted in the design of a group of 4-quinolone-3-glyoxylic acids [49] that retained the coplanarity of DKA functional groups. A potent compound from this original study contained only a β-ketone functional group and a carboxylic acid functional group, which were coplanar, and showed a 1.6 μM IC_50 _value in a strand transfer assay. Derivatives of this parent compound exhibited up to a 7.2 nM IC_50 _value in strand transfer assays and a 0.9 nM EC_50 _in an antiviral assay. This activity proved that a monoketo motif could be an efficacious alternative to the accepted DKA. A 2005 license agreement between Japan Tobacco and Gilead Sciences led to the clinical development of GS-9137 (a.k.a. elvitegravir) [Figure 1, [43]], a quinolone carboxylic acid strand-transfer specific inhibitor that displayed an IC_50 _of 7 nM against IN and an antiviral EC_90 _of 1.7 nM in the presence of NHS. In terms of pharmacokinetics (Additional file 1), in rat and dog elvitegravir displayed a 34% and 30% bioavailability, a 2.3 h and 5.2 h half-life, and a 8.3 mL/min/kg and 17 mL/min/kg clearance, respectively. Interestingly though, its half-life in human was shown to increase from 3 hours when dosed alone to 9 hours when boosted with the protease inhibitor, ritonavir [53]. Similarly, its bioavailability increased 20-fold when administered in combination with ritonavir. These observations back a valid argument that elvitegravir may become a second-generation IN inhibitor, in that its significantly improved pharmacokinetic profile when boosted may increase patient compliance by allowing a simple once daily treatment (raltegravir is administered twice daily). Similar to raltegravir, though, elvitegravir has been shown to provoke T66I and E92Q viral resistance mutations, as well as substitutions of amino acids flanking raltegravir-induced substitution sites (Q146P and S147G) [54].

### GSK-364735

In studies to develop follow-on analogs of S-1360, the two involved groups jointly discovered a novel lead naphthyridinone, GSK-364745 (Figure 1). This compound contains a hydrophobic fluorobenzyl substituent flexibly linked to a chelatable quinolone region. GSK-364735 inhibited IN in an *in vitro *strand transfer assay with an IC_50 _of 8 nM, and it showed an antiviral EC_90 _value of 40 nM in MT-4 cells in the presence of 20% NHS. Acceptable pharmacokinetics were achieved, with bioavailabilities of 42%, 12%, and 32%; half-lives of 1.5 h, 1.6 h, and 3.9 h; and clearances of 3.2 mL/min/kg, 8.6 mL/min/kg, and 2 mL/min/kg in rat, dog, and rhesus monkey, respectively (Additional file 1). However, when tested against mutant viruses, the compound exhibited greatly decreased activity – 17-fold reduction against T66K, 210-fold reduction against Q148K, 73-fold reduction against Q148R, and 23-fold reduction against N155S [55].

### BMS-707035

A pyrimidine carboxamide similar in structure to raltegravir was recently propelled into Phase II clinical trials by a separate group. This compound was different from raltegravir in that raltegravir's 1,3,4-oxadiazole group was substituted with a cyclic sulfonamide moiety (Figure 1), but its *in vitro *potency was similar with an IC_50 _value of 20 nM. However, multiple mutations were almost immediately observed to have occurred in viral response to treatment with BMS-707035, which included V75I, Q148R, V151I, and G163R [32]. Unfortunately, the severity of resistance conferred by each of these mutations has not been disclosed, nor have pharmacokinetic properties of the drug. What is known, however, is that the drug did not last long in Phase II trials, and testing was abruptly terminated in early 2008 [56]. An explanation of the termination of the trial has not been publicly provided.

## Novel me-too classes

### Dihydroxypyrimidine-4-carboxamides

**Figure 3:**
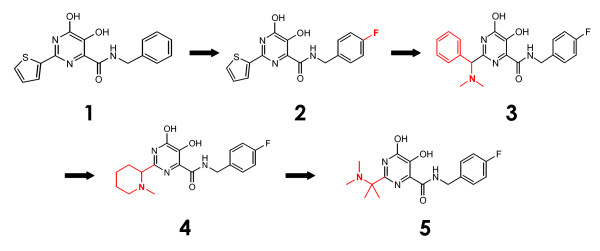
**The evolution of dihydroxypyrimidine-4-carboxamides**.

Soon after promising clinical data regarding the progress of MK-0518 became available, a novel DKA-related class of IN inhibitory compounds (Figure 3, Additional file 1) was developed through screening of inhibitors of HCV polymerase, which demonstrates a high degree of structural similarity to IN [31]. Specifically, IN and HCV polymerase possess a similar active site amino acid geometry, and both utilize two magnesium ions in their catalysis. A class of dihydroxypyrimidine carboxamides was derived as HCV polymerase inhibitors from DKAs, and they were found to exhibit improved drug-like properties and correct Mg^2+ ^binding geometry. Most of these compounds were inactive against IN, but a substitution of the free carboxylic acid with a benzyl amide yielded compound **1**, with nanomolar IN inhibitory activity in enzymatic assays. Compound **1 **showed a decent pharmacokinetic profile, with a bioavailability of 15%, plasma clearance of 5 mL/min/kg, and a half-life of 3 hours. Further structure activity relationship (SAR) studies upon the amide moiety of 1 led to the identification of a superior *para*-fluorobenzyl substituent (compound 2). Compound 2 exhibited an IC_50 _of 10 nM in the enzymatic assay, as well as an improved oral bioavailability in rats of 29%. However, both compounds 1 and 2 were inactive in cell-based assays, due to poor solubility, poor cell permeability, and significant plasma protein binding [31].

This group pushed on in their search for raltegravir me-too drugs with further SAR studies upon the above *N*-alkyl hydroxypyrimidinone lead compounds (Figure 3). As a benzyl amide substitution of a free carboxyl instilled nanomolar activity upon said compounds, a library of over 200 different amide modifications was synthesized and screened for inhibitory potency [57]. A 4-fluoro-substituted benzene was shown to be optimal for IN inhibition, with an IC_50 _value in enzymatic assays of 10 nM. However, though compounds optimized in this fashion were active in the enzymatic assay, they lacked potency in cell based assays. The thiophene ring in the 2-position of the pyrimidine core was shown to have little effect upon the interaction of the compound with IN, and so this position was chosen for more dramatic changes influencing physiochemical properties of inhibitors. Introduction of a basic group to a 2-benzyl derivative resulted in increased cell permeability and inhibition of viral replication in the presence of fetal bovine serum (FBS) with a CIC_95 _of 300 nM (compound 3). This compound showed an oral bioavailability of 59% and 93%, a half-life of 1.73 h and 6.78 h, and a plasma clearance of 14 mL/min/kg and 0.5 mL/min/kg in rats and dogs, respectively. However, weak activity in the presence of 50% NHS exposed the mobile nature of chosen 2-position substituents. In response the phenyl group at this position was removed and the NH methylated, to confer reduced lipophilicity (and reduced plasma protein binding) but maintain the presence of the mandatory amino group. Compound 4 was thus born, exhibiting a 95% human plasma protein binding and a 400 nM CIC_95 _in the presence of 50% NHS. Pharmacokinetics of compound 4 included an oral bioavailability of 27% and 90%, a half-life of 0.43 h and 6.0 h, and a plasma clearance of 75 mL/min/kg and 2 mL/min/kg in rats and dogs, respectively. Separately, smaller acyclic amines were substituted into the 2 position and similarly assayed for activity [57]. It was found that a dimethylaminomethyl substituent separated by an sp^3^-carbon spacer bestowed significant cell based potency, at a CIC_95 _of 78 nM in 50% NHS (compound 5). In rats, dogs, and monkeys, compound **5 **had a prolonged plasma half-life (2.1, 4.8, and 1.9 h, respectively), moderate to low clearance (16, 1.9, and 15 mL/min/kg, respectively) and moderate to excellent oral bioavailability (28%, 100%, and 61%, respectively) [57].

### N-methylpyrimidones

**Figure 4:**
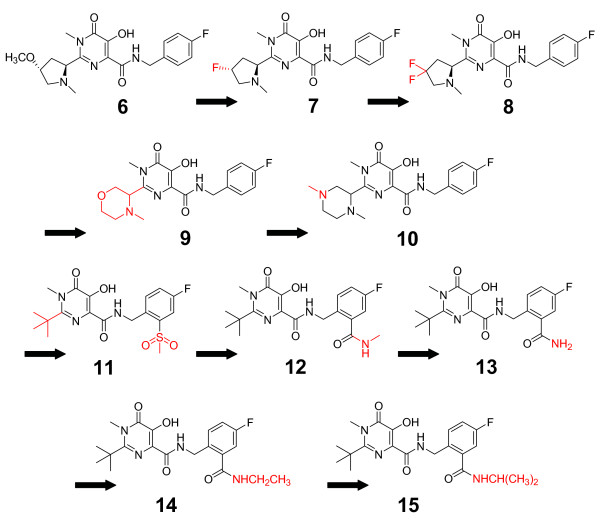
**The evolution of N-methylpyrimidones**.

To improve cell-based potency and bioavailability of the above molecules, this group began to study the effect of methylation of their N-1 pyrimidine nitrogens (Figure 4, Additional file 1). The rationale for this decision was based upon their discovery that the amine contained in the ring must occupy the benzylic position with respect to the pyrimidine and that small alkyl groups are preferred on the nitrogen of the saturated heterocycle [57]. A methyl group was initially scanned on the pyrrolidine ring, and substitution on position 4 gave the best enzymatic activity. Substitution of the free hydroxyl group of a resulting *trans*-4-hydroxy pyrrolidine with a methoxy substituent produced potent activity (compound 6) in both *in vitro *(IC_50 _= 180 nM) and cell-based assays (CIC_95 _= 170 nM in 50% NHS) [58]. From here the group tested other substitutions, of which a fluorine (compound 7 – CIC_95 _= 250 nM) or a difluoro derivative (compound 8 – CIC_95 _= 170 nM) were well accepted. Activity was found to be further augmented by substituting a six-membered derivative in position 2 of the pyrimidine, and the morpholine derivative 9 and piperidine derivative 10 displayed slightly improved cell-based potencies (100 nM and 190 nM CIC_95 _in 50% NHS, respectively). In terms of pharmacokinetics, the morpholine derivative 9 was the most ideal candidate for further testing, with bioavailabilities of 92%, 100%, and 53%; half-lives of 1.5 h, 10 h, and 1.4 h; and plasma clearance rates of 22 mL/min/kg, 3 mL/min/kg, and 14 mL/min/kg in rat, dog, and rhesus monkey, respectively [58].

A further optimization study analyzed the enzymatic and pharmacokinetic implications of a different, ^*t*^butyl substitution at the C-2 position of the pyrimidine scaffold of the above compounds [Figure 4, [59]]. Further introduction of a benzylamide to the right side of the scaffold proved necessary for activity in serum conditions. Multiple derivatives were designed using the *N*-methyl pyrimidone scaffold, including a sulfone (compound 11) and an *N*-methyl amide (compound 12) that showed CIC_95_s of 20 nM and 10 nM in 50% NHS, respectively. This encouraging data inspired further substitutions of the 2-*N*-methyl carboxamide, for optimization of pharmacokinetic behavior. An unsubstituted amide 13 exhibited a promising inhibitory profile (IC_50 _= 20 nM in enzymatic assay, CIC_95 _= 10 nM in 50% NHS), prompting multiple further substitutions of the *N*-methyl residue with an *N*-ethyl (compound 14) and an ^*i*^*N*-propyl (compound 15). The pharmacokinetic profiles of 11, 12, and 13 were not optimal (Additional file 1), and none of these substitutions were beneficial in this respect. Bioavailability was 17%, 18%, and 23%; half-life was 1.8 h, 1.6 h, and 3.6 h; and plasma clearance was 37 mL/min/kg, 24 mL/min/kg, and 55 mL/min/kg in rat for 11, 12, and 13, respectively [59].

### Dihydroxypyrido-pyrazine-1,6-diones

**Figure 5:**
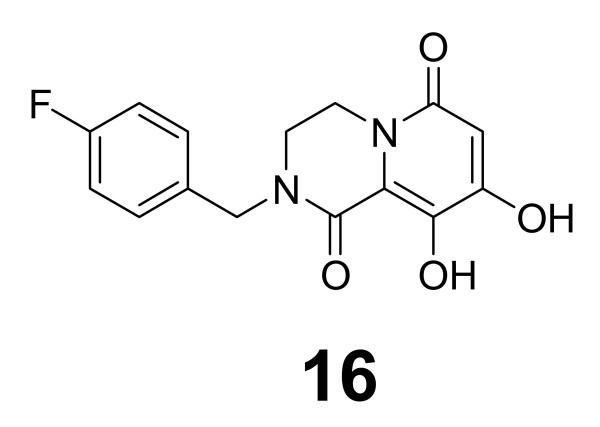
**Dihydroxypyrido-pyrazine-1,6-dione representative example**.

Parallel to the above N-methylpyrimidone studies, the same group was working toward optimization and cyclic constraint of the dihydroxypyrimidine-4-carboxamide amide side chain, yielding a novel class of dihydroxypyridopyrazine-1,6-dione compounds [Figure 5, [60]]. Coplanarity of the amide carbonyl group in the constrained ring with respect to the dihydroxypyridinone core and a resulting limitation of flexibility of the 4-fluorobenzyl side chain (compound 16) were shown through molecular modeling to be essential for inhibitory activity. Compound 16 inhibited IN strand transfer *in vitro *at an IC_50 _of 100 nM and HIV replication in cell culture at a CIC_95 _of 310 nM, with little cytotoxicity. Limited pharmacokinetic data has been provided for this class of compounds, but compound 16 was shown to have a 69% oral bioavailability in rats, and plasma concentrations were maintained between 0.64 and 0.50 μM from the second to the twenty-fourth hour (Additional file 1). There was concern about the dihydroxypyrimidone core and its metabolites irreversibly associating with liver microsomal proteins, but only a non-significant level (<50 pmol equiv/mg/60 min) of interaction was observed [60].

### Bicyclic pyrimidones

**Figure 6:**
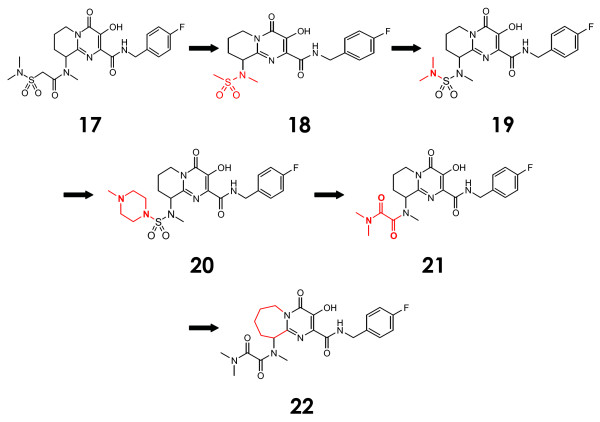
**The evolution of bicyclic pyrimidones**.

Recently, the aforementioned importance of a β-amino substituent in the 2-position of the pyrimidine scaffold and the beneficial effect of the 1*N*-methylation were exploited in a systematic constraint of the 1*N*-methyl on the 1*N*-methylpyrimidinone scaffold (Figure 6, Additional file 1). With unsubstituted benzylmethylamine derivatives showing nanomolar enzymatic inhibition profiles similar to those of derivatives with saturated ring side chains (though little inhibition of viral replication in cell culture), it was decided that the 2-*β*-nitrogen would be modified to optimize physiochemical properties of pyrimidone compounds [61]. For example, introduction of a sulfonamide (compound 17) resulted in a low shift in activity in serum conditions, suggesting an increased level of cell permeability. The (*R*)-17 enantiomer displayed a 7 nM enzymatic IC_50 _value, a 31 nM CIC_95 _in 50% NHS (two-fold more potent than its (*S*)-17 enantiomer contemporary), and acceptable pharmacokinetics including a 17% bioavailability and 55 mL/min/kg plasma clearance in rat. Sulfonamide derivatives showed similarly decent profiles (compound 18 = 12 nM IC_50 _against strand transfer, 86 nM CIC_95 _in cells in 50% NHS, and a 47% bioavailability and 48 mL/min/kg plasma clearance in rats). However, an even more significant improvement in potency occurred upon changing the sulfonamide moiety to a tetrasubstituted sulfamide (compound 19). The (*R*)-19 enantiomer inhibited IN with an IC_50 _value and a CIC_95 _value of 7 nM and 44 nM, respectively, but pharmacokinetics (9% bioavailability in rhesus monkey) were inadequate. Introduction of a more polar *N*-methylpiperazine (compound 20), however, produced a compound whose (S)-20 enantiomer inhibited IN at a CIC_95 _of 6 nM in cell culture in the presence of 50% NHS. This compound was much more stable toward glucuronidation than its sulfamide counterpart, but low bioavailability and high plasma clearance in rats and dogs neutralized its promise. It was hence necessary to make use of other nitrogen functionalizations in order to optimize these pharmacological properties. The substitution of ketoamides and enlarged rings (compounds 21 and 22, respectively) resulted in potent inhibition of IN in cell based assays and much improved pharmacokinetics. The (*S*)-enantiomers of both compounds achieved CIC_95_s of 43 nM and 13 nM in cell culture, respectively, as well as moderate pharmacologic properties in rats, dogs, and (compound (*S*)-22 only) monkeys [61].

### Pyrrolloquinolones

**Figure 7:**
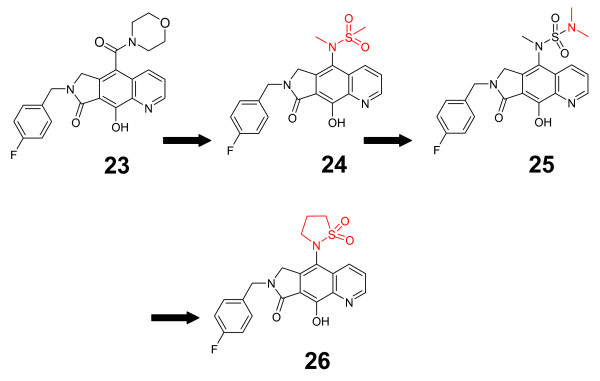
**The evolution of pyrrolloquinolones**.

A different group has recently built upon their prior optimization of the clinically efficacious L870,810 [62,63] by varying C5 substituents within their compounds' tricyclic scaffolds (Figure 7, Additional file 1). They originally developed the tricyclic scaffold to provide a pre-organized, energetic improvement to L870,810's unfavorable energy consumption upon rotational conversion from free state to bound state, leading to a more soluble and potent compound **23 **[62]. In their recent work, C5-amino derivatives were prepared and assayed for improvement in strand transfer inhibitory potency and pharmacokinetics, due to their projected higher stability against hydrolysis than analogous carbamates or sulfamates [64]. The most promising leads turned out to be a C5 sulfonamide (compound 24), a C5 sulfonylurea (compound 25), and a C5 sultam (compound 26). Compounds 24 and 25 retained potency in the presence of serum albumin and α-1 acidic glycoproteins, while 26 was negatively affected. Though the sultam 26 showed a lower IC_50 _than the sulfonamide 24 and sulfonylurea 25 in enzymatic assays (13 nM as opposed to 28 nM and 62 nM, respectively), it lacked potency in cell culture in 50% NHS (EC_50 _49 nM as opposed to 11.4 nM and 8.4 nM, respectively). It is important to note that raltegravir showed an EC_50 _value of 16 nM in cell culture in the presence of 50% NHS. Compound 26 was additionally lacking in bioavailability in both rat (4%) and dog (8%). However, compounds 24 and 25 showed slightly more promising profiles, with bioavailabilities of 15%/13% and 45%/16% and half-lives of 1.1 h/0.9 h and 4.9 h/4.5 h in rat and dog, respectively [64]. This study exemplified the importance of rigidifying inhibitor pharmacophores in terms of conferring favorable potency and pharmacokinetic properties.

### Validation of resistance profiles of me-too raltegravir analogues

**Figure 8:**
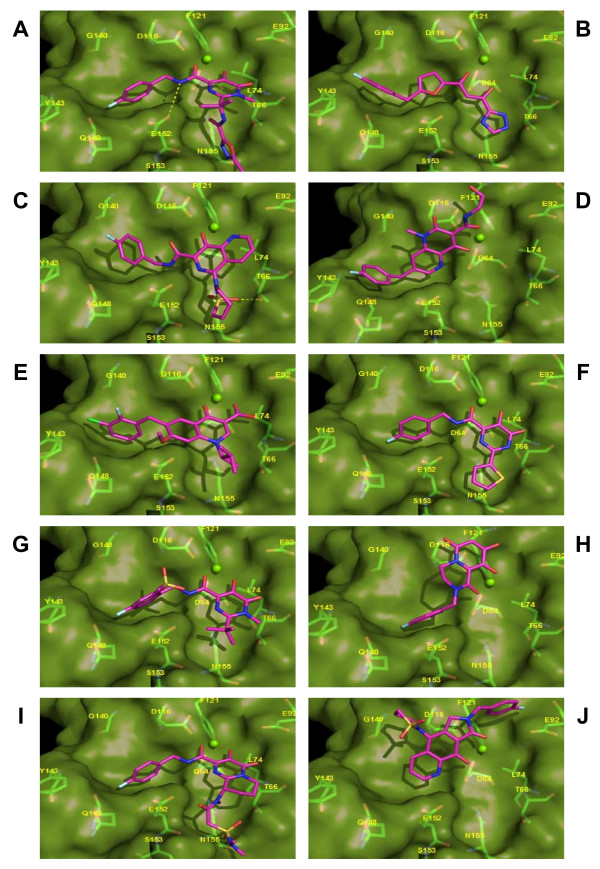
**Docking poses of selected HIV-1 integrase inhibitors upon the 1BL3 IN crystal structure**. A, MK-0518; B, S-1360; C, L870,810; D, GSK-364735; E, GS-9137; F, compound 2; G, compound 11; H, compound 16; I, compound 17; J, compound 26.

Though there is minor variation in the *in vitro *activity of the above me-too IN inhibitors, their structures, mechanisms of action, and pharmacokinetics are highly similar. We believe that the development of me-too compounds may yield a relatively low amount of clinical success due to their similarities, and also due to the fact that nearly identical resistance profiles will be evoked by their application. However, we would like to note that it is definitely possible for a raltegravir me-too analog to evolve into a second-generation IN inhibitor. To further elucidate our viewpoint, we utilized the molecular docking program GOLD version 3.2 to conduct a docking study, using both the X-ray determined structure of 1BL3 IN complexed with an Mg^2+ ^ion, and a collection of significant, above-described me-too compounds (Figure 8); for a detailed procedure, see [65]. We propose that residues essential to the compounds' interaction with IN will obviously be prime candidates for resistance mutation. Furthermore, we hypothesize that the test of time will show that all of these me-too inhibitors will probably exhibit highly similar resistance profiles. As raltegravir has undergone extensive resistance profiling since the inception of its clinical employment (Table 1), we first compared our predicted interaction residues (Figure 8) to these experimental profiles, as a validation of the reliability of our technique. We found that five of our predicted interaction residues (T66, E92, Y143, Q148, and N155) have been already observed to confer a range of anywhere from 5- to 35-fold resistances to raltegravir inhibition of viral replication, respectively [66-69]. We also saw that raltegravir makes direct interactions with the three residues encompassing the IN catalytic DDE motif (D64, D116, and E152), including a hydrogen bond with the glutamate. With this technique corroboration in hand, we decided to similarly predict the interaction residues of raltegravir's progenitors and a few me-too analogues, in order to provide evidence for our assertion that these compounds will ultimately experience a low probability of success in viral eradication, due to their generation of identical resistance profiles. As S-1360 was the first clinical IN inhibitor candidate, we thought it would be interesting to evaluate the similarity between its predicted interaction profile with 1BL3 (Figure 8) and that of raltegravir. We found that an identical interaction occurs between the two drugs and IN (D64, T66, D116, Y143, Q148, E152, and N155), but predicted an additional interaction of raltegravir with E92. This observation has been verified in clinical experimental resistance profiling, as mutation of E92 has not been observed for S-1360, but the E92Q mutation has conferred up to a 7-fold viral resistance to raltegravir [25,26,70]. We next observed the interaction profile of 1BL3 with L870,810 (Figure 8), as this is the naphthyridine carboxamide compound that directly led to the development of pyrimidinone carboxamides. We found that L870,810 and raltegravir similarly interacted with D64, T66, D116, Q148, E152, and N155. However, we saw here that only raltegravir interacted with E92. Though this residue has been observed to be mutated to a glutamine in response to L870,810 treatment, the mutation has conferred at most only a 2-fold resistance to the drug, while the same mutation confers up to a 7-fold resistance to raltegravir (Table 1) [29,71]. The fact that we did not observe a significant interaction between L870,810 and E92 in our docking study further confirms the relatively decreased importance of this residue in viral resistance to the compound. Along the same lines, we did see an interaction of L870,810 with V151, an interaction that was not present in our docking of raltegravir. In clinical experimental resistance profiling, the V151I mutation has been observed to confer up to an 18-fold resistance to L870,810, while the same mutation had a negligible effect on viral resistance to raltegravir (Table 1) [29,71]. The highly homologous naphthyridine carboxamide candidate, L870,812, has shown an interaction profile virtually identical to that of L870,810 in our docking study, and experimental resistances obtained in clinical observation have been identical as well [29,71]. As elvitegravir (GS-9137) and GSK-364735 have already been shown to exhibit near identical resistance profiles to raltegravir (Table 1) [67,71-73], we next used our docking technique to attempt to effectively predict these interactions (Figure 8). For GSK-364735, we were able to predict the interaction with IN residues Y143 and Q148, as well as the three members of the DDE motif. We then predicted that, similar to raltegravir, elvitegravir interacts with T66, E92, Y143, Q148, and the D116 and E152 of the DDE motif. We also saw that elvitegravir interacts with G140, and the G140S mutation has been shown to be associated with a 4-fold viral resistance to the drug, while the same mutation confers only a 1.6-fold resistance to raltegravir (Table 1). Again, the fact that we did not observe a significant interaction between raltegravir and G140 in our docking study further confirms the relatively decreased importance of this residue in viral resistance to raltegravir, but rather its nature of compensation for more meaningful mutations, such as Q148H.

**Table 1:** Effect of single mutations on IN sensitivity to clinically tested inhibitors.

Mutation	S-1360	L870, 810	MK-0518	GS-9137	GSK-364735
T66I	++	+	++	+++	+

L68V			+	+	

L68I			+	+	

V72I		+		++	

L74M	++++	+	+	++	

E92Q	++	+	++	+++	++

Q95K		++		++	

F121Y	+++	++	++	+++	+++

T124A	+				+

T125K		+	+	+	

E138K	+	+	+	+	

G140S	++		+	++	

P145S	++				+

Q146R	++	+		+++	+

S147G		++	+	++	

Q148H	+++		+++	++	

Q148R	++++		+++	+++++	++++

Q148K	++++		+++	++++	+++++

V15II		+++	+	++	

S153Y	++		+	++	+

N155H	++		+++	+++	

N155S	+++	++	++		+++

E157Q				++	

R263K				++	

E92Q/N155H			++++	++++	

F121Y/T125K			++	++++	

G140S/Q148H			+++++	+++++	

V72I/F121Y/T125K/V151I		+++++		+++++	

Fold change in potency compared to wild type: + <2, ++ 2–10, +++ 11–50, ++++ 51–100, +++++ >100

## Prediction of future me-too resistance profile similarities

With the above data significantly validating the reliability of our docking technique, we moved forward with the prediction of resistance profiles of selected me-too raltegravir analogues (Figure 8). Here, we will describe the interactions of one of the most potent (in terms of *in vitro *IC_50 _inhibition of IN) compounds from each of the above-described classes of me-too inhibitors with the 1BL3 IN crystal structure. The dihydroxypyrimidine-4-carboxamide compound 2 exhibited an IC_50 _value of 10 nM against IN [31]. However, our predicted interaction profile for this compound shows that it will most likely be ineffective against raltegravir-resistant viruses. We show that compound 2 interacts with 1BL3 IN residues D64, T66, E92, D116, Q148, E152, S153, and N155 – virtually the exact binding pocket as raltegravir. The N-methylpyrimidone compound 11 exhibited an IC_50 _value of 20 nM against IN [58]. Our predicted interaction profile for this compound encompasses the 1BL3 residues D64, T66, E92, D116, G140, Y143, Q148, E152, and N155 – virtually the exact binding pocket as raltegravir. The dihydroxypyrimido-pyrazine-1,6-dione compound 16 had a moderate IC_50 _value of 100 nM against IN [60]. Our predictive docking procedure calculated an interaction profile including IN residues D64, E92, D116, and Q148. E92Q and Q148R mutations have already been observed to confer 7-fold and 35-fold resistances to raltegravir, respectively. The bicyclic pyrimidone compound **17 **has displayed a potent IC_50 _value of 7 nM against IN *in vitro *[61]. However, our predicted interaction profile implicates the 1BL3 residues D64, T66, E92, D116, Y143, Q148, and E152 as contact points. This is virtually the same binding pocket as that of raltegravir. Finally, the pyrrolloquinolone compound 26 has exhibited an IC_50 _value of 13 nM against IN [64]. Again however, we show that this compound will interact with IN in a considerably similar manner to that of raltegravir, contacting residues D64, E92, D116, G140, and E152. If our predictions prove to be correct, these candidate drugs will probably fail to replace raltegravir. Though me-too evolution into a new blockbuster drug is always a possibility, the above IN me-too drugs appear to have a small chance of improving the clinical outlook of HIV patients with raltegravir-resistant viral strains.

## Conclusion

As me-too drugs have been historically shown to be minimally progressive in terms of improvement of disease prognosis, their lack of utility is exemplified in the case of HIV. A plethora of polymorphic resistance mutations have almost instantly arisen in response to both raltegravir and the purported second-generation IN inhibitor, elvitegravir [74]. It is clear to see that the virus is capable of eventually avoiding interaction with many a once potent inhibitor, and attempts at recreating these original interactions will most likely fall victim to the same mode of viral escape. Although some pharmacokinetic properties may be optimized through me-too drug development research, and some profitable drugs may be cleared for marketing, the long term efficacy of most of these drugs will likely be susceptible to the ever present mutational ultra-competence of HIV. As stated earlier, there is a thin line between drug development and me-too spawning. Simple pharmacokinetic improvement can drastically augment the daily lives of patients and the quarterly profits of companies, but the simple fact remains that HIV will most likely not be eliminated by a 2% increase in oral bioavailability. Dramatically diverse classes of molecules look to be required for inhibition of viral enzymes in a long term fashion. Thus, in our eyes, the only hope for complete viral eradication is innovation.

## Competing interests

The authors declare that they have no competing interests.

## Authors' contributions

ES and NN conceived the article. ES wrote the article. SO performed docking studies. KR provided information regarding novel me-too IN inhibitor classes. All authors have read and approved the final manuscript.

## Supplementary Material

Additional file 1 Table S1. Classification, activity, and pharmacokinetic data for IN inhibitory compounds described herein. Click here for file
