# Raltegravir, elvitegravir, and metoogravir: the birth of "me-too" HIV-1 integrase inhibitors

**DOI:** 10.1186/1742-4690-6-33

**Published:** 2009-04-08

**Authors:** Erik Serrao, Srinivas Odde, Kavya Ramkumar, Nouri Neamati

**Affiliations:** 1 Department of Pharmacology and Pharmaceutical Sciences, University of Southern California, School of Pharmacy, 1985 Zonal Avenue, Los Angeles, CA 90089, USA

## Abstract

Correction to Erik Serrao, Srinivas Odde, Kavya Ramkumar and Nouri Neamati: Raltegravir, elvitegravir, and metoogravir: the birth of "me-too" HIV-1 integrase inhibitors. *Retrovirology *2009, 6:25. Since the recent publication of our article (Neamati, *Retrovirology *2009, 6:25), we have noticed an error which we would like to correct and we would like to apologise to the readers for this mistake.

## Correction

The structure of BMS-707035 has not been publicly released. In Figure one on page 2 and a discussion on page 5 of our published paper [1], we mistakenly assigned a structure and activity data to BMS-707035. The structure and activity data that we mentioned actually belong to a different compound from the same group. The resistance pattern of BMS-707035 is "overlapping" rather than identical to raltegravir. Please see the following references [2,3] for a detailed description of their development of pyrimidine carboxamides.
