# Supplementary material for: Raltegravir, elvitegravir, and metoogravir: the birth of "me-too" HIV-1 integrase inhibitors
Source: Retrovirology. 2009 Mar 5;6:25. doi: 10.1186/1742-4690-6-25 (PMC2660292; doi:10.1186/1742-4690-6-25)
Supplement: Additional file 1 — Table S1. Classification, activity, and pharmacokinetic data for IN inhibitory compounds described herein. [file 1742-4690-6-25-S1.doc]

| **COMPOUND** | **CLASS** | **ENZYMATIC ASSAY INHIBITION** | **CELL-BASED INHIBITION** | **PHARMACOKINETIC DATA** |
| --- | --- | --- | --- | --- |
| **Elvitegravir,**  (Sato, 2006)  **Gilead Sciences** | **4-quinolone-3-carboxylic acid** | IC_50_ = 7 nM | EC_50_ = 0.7 nM  EC_90_ = 1.7 nM (serum-adjusted) | Rat: F = 34%  T_1/2_ = 2.3 h  Cl = 8.3 mL/min/kg |
|  |  |  |  | Dog: F = 29.6%  T_1/2_ = 5.2 h  Cl = 17 mL/min/kg |
| **GSK-364735,**  (Garvey 2008)  **Glaxo Smith Kline** | **1,6-naphthyridinone carboxamide** | IC_50_ = 8 nM | EC_50_ = 1.2 nM  EC_90_ = 42 nM (serum-adjusted) | Rat: F = 42%  T_1/2_ = 1.5 h  Cl = 3.2 mL/min/kg |
|  |  |  |  | Dog: F = 12%  T_1/2_ = 1.6 h  Cl = 8.6 mL/min/kg |
|  |  |  |  | Rhesus:  F = 32%  T_1/2_(*i.v.*) = 3.9 h  Cl = 2 mL/min/kg |
| **1**, (Summa,2006)  **Merck** | **Dihydroxy-pyrimidine-4-carboxamide** | IC_50_ = 80 nM | IC_95_ > 10 μM  (10% FBS) | Rat: F = 15%  T_1/2_(*i.v.*) = 3 h  Cl = 5 mL/min/kg |
| **2**, (Summa,2006)  **Merck** | **Dihydroxy-pyrimidine-4-carboxamide** | IC_50_ = 10 nM | IC_95_ > 10 μM  (10% FBS) | Rat: F = 29%  T_1/2_(*i.v.*) = 1.3 h  Cl = 11 mL/min/kg |
| **3**, (Pace,2007)  **Merck** | **Dihydroxy-pyrimidine-4-carboxamide** | IC_50_ = 200 nM | IC_95_ = 310 nM  (10% FBS) | Rat: F = 59%  T_1/2_(*i.v.*) = 1.73 h  Cl = 14 mL/min/kg |
|  |  |  |  | Dog: F = 93%  T_1/2_(*i.v.*) = 6.78 h  Cl = 0.5 mL/min/kg |
| **4**, (Pace,2007)  **Merck** | **Dihydroxy- pyrimidine-4-carboxamide** | IC_50_ = 200 nM | IC_95_ = 140 nM  (10% FBS)  IC_95_ = 400 nM  (50% NHS) | Rat: F = 27%  T_1/2_(*i.v.*) = 0.43 h  Cl = 75 mL/min/kg |
|  |  |  |  | Dog: F = 90%  T_1/2_(*i.v.*) = 6 h  Cl = 2 mL/min/kg |

| **5**, (Pace,2007)  **Merck** | **Dihydroxy- pyrimidine-4-carboxamide** | IC_50_ = 50 nM | IC_95_ = 60 nM  (10% FBS)  IC_95_ = 78 nM  (50% NHS) | Rat: F = 28%  T_1/2_(*i.v.*) = 2.1 h  Cl = 16 mL/min/kg |
| --- | --- | --- | --- | --- |
|  |  |  |  | Dog: F = 100%  T_1/2_(*i.v.*) = 4.8 h  Cl = 1.9 mL/min/kg |
|  |  |  |  | Rhesus: F = 61%  T_1/2_(*i.v.*) = 1.9 h  Cl = 15 mL/min/kg |
| **6**, (Gardelli,2007)  **Merck** | **N-methyl-pyrimidone** | IC_50_ = 180 nM | IC_95_ = 150 nM  (10% FBS)  IC_95_ = 170 nM  (50% NHS) | Rat: F = 79%  T_1/2_(*i.v.*) = 1.5 h  Cl = 19 mL/min/kg |
| **7**, (Gardelli,2007)  **Merck** | **N-methyl- pyrimidone** | IC_50_ = 20 nM | IC_95_ = 130 nM  (10% FBS)  IC_95_ = 250 nM  (50% NHS) | Rat: F = 100%  T_1/2_(*i.v.*) = 2 h  Cl = 12 mL/min/kg |
| **8**, (Gardelli,2007)  **Merck** | **N-methyl- pyrimidone** | IC_50_ = 30 nM | IC_95_ = 30 nM  (10% FBS)  IC_95_ = 170 nM  (50% NHS) | Rat: F = 93%  T_1/2_(*i.v.*) = 0.6 h  Cl = 11 mL/min/kg |
|  |  |  |  | Dog: F = 100%  T_1/2_(*i.v.*) = 6 h  Cl = 5 mL/min/kg |
| **9**, (Gardelli,2007)  **Merck** | **N-methyl- pyrimidone** | IC_50_ = 60 nM | IC_95_ = 60 nM  (10% FBS)  IC_95_ = 100 nM  (50% NHS) | Rat: F = 92%  T_1/2_(*i.v.*) = 1.5 h  Cl = 22 mL/min/kg |
|  |  |  |  | Dog: F = 100%  T_1/2_(*i.v.*) = 10 h  Cl = 3 mL/min/kg |
|  |  |  |  | Rhesus: F = 53%  T_1/2_(*i.v.*) = 1.4 h  Cl = 14 mL/min/kg |
| **10**, (Gardelli,2007)  **Merck** | **N-methyl- pyrimidone** | IC_50_ = 100 nM | IC_95_ = 250 nM  (10% FBS)  IC_95_ = 190 nM  (50% NHS) | Rat: F = 62%  T_1/2_(*i.v.*) = 0.7 h  Cl = 21 mL/min/kg |

| **11**, (Di Francesco,2008)  **Merck** | **N-methyl-pyrimidone** | IC_50_ = 20 nM | IC_95_ = 10 nM  (10% FBS)  IC_95_ = 20 nM  (50% NHS) | Rat: F = 17%  T_1/2_(*i.v.*) = 1.8 h  Cl = 37 mL/min/kg |
| --- | --- | --- | --- | --- |
| **12**, (Di Francesco,2008)  **Merck** | **N-methyl-pyrimidone** | IC_50_ = 20 nM | IC_95_ = 10 nM  (10% FBS)  IC_95_ = 10 nM  (50% NHS) | Rat: F = 18%  T_1/2_(*i.v.*) = 1.6 h  Cl = 24 mL/min/kg |
| **13**, (Di Francesco,2008)  **Merck** | **N-methyl-pyrimidone** | IC_50_ = 20 nM | IC_95_ = 10 nM  (10% FBS)  IC_95_ = 10 nM  (50% NHS) | Rat: F = 23%  T_1/2_(*i.v.*) = 3.6 h  Cl = 55 mL/min/kg |
| **14**, (Di Francesco,2008)  **Merck** | **N-methyl-pyrimidone** | IC_50_ = 70 nM | IC_95_ = 20 nM  (10% FBS)  IC_95_ = 20 nM  (50% NHS) | Rat: F = 30%  T_1/2_(*i.v.*) = 2.6 h  Cl = 21 mL/min/kg |
| **15**, (Di Francesco,2008)  **Merck** | **N-methyl-pyrimidone** | IC_50_ = 90 nM | IC_95_ = 60 nM  (10% FBS)  IC_95_ = 60 nM  (50% NHS) | Rat: F = 44%  T_1/2_(*i.v.*) = 1.6 h  Cl = 35 mL/min/kg |
| **16**, (Wai,2007)  **Merck** | **Dihydroxypyrido-pyrazine-1,6-dione** | IC_50_ = 100 nM | IC_95_ = 310 nM  (10% FBS)  IC_95_ = 310 nM  (50% NHS) | Rat: F = 69% |
| **17**, (Muraglia,2008)  **Merck** | **Bicyclic pyrimidone** | IC_50_ = 7 nM | IC_95_ = 16 nM  (10% FBS)  IC_95_ = 31 nM  (50% NHS) | Rat: F = 17%  Cl = 55 mL/min/kg |

| **18**, (Muraglia,2008)  **Merck** | **Bicyclic pyrimidone** | IC_50_ = 12 nM | IC_95_ = 26 nM  (10% FBS)  IC_95_ = 86 nM  (50% NHS) | Rat: F = 47%  Cl = 48 mL/min/kg |
| --- | --- | --- | --- | --- |
| (*R*)**-19**, (Muraglia,2008)  **Merck** | **Bicyclic pyrimidone** | IC_50_ = 7 nM | IC_95_ = 16 nM  (10% FBS)  IC_95_ = 44 nM  (50% NHS) | Rat: F = 63%  Cl = 1.8 mL/min/kg |
|  |  |  |  | Dog: F = 42%  Cl = 3.8 mL/min/kg |
|  |  |  |  | Rhesus: F = 9%  Cl = 27 mL/min/kg |
| (*S*)-**20**, (Muraglia,2008)  **Merck** | **Bicyclic pyrimidone** | IC_50_ = 7 nM | IC_95_ = 6 nM  (10% FBS)  IC_95_ = 6 nM  (50% NHS) | Rat: F = 15%  Cl = 98 mL/min/kg |
|  |  |  |  | Dog: Cl = 23 mL/min/kg |
| (*S*)-**21**, (Muraglia,2008)  **Merck** | **Bicyclic pyrimidone** | IC_50_ = 12 nM | IC_95_ = 33 nM  (10% FBS)  IC_95_ = 43 nM  (50% NHS) | Rat: F = 47%  T_1/2_(*i.v.*) = 14 h  Cl = 11 mL/min/kg |
|  |  |  |  | Dog: F = 39%  T_1/2_(*i.v.*) = 1.7 h  Cl = 15 mL/min/kg |
| (*S*)-**22**, (Muraglia,2008)  **Merck** | **Bicyclic pyrimidone** | IC_50_ = 12 nM | IC_95_ = 10 nM  (10% FBS)  IC_95_ = 13 nM  (50% NHS) | Rat: F = 64%  T_1/2_(*i.v.*) = 7.4 h  Cl = 7 mL/min/kg |
|  |  |  |  | Dog: F = 61%  T_1/2_(*i.v.*) = 2.1 h  Cl = 7.5 mL/min/kg |
|  |  |  |  | Rhesus: F = 31%  T_1/2_(*i.v.*) = 2.6 h  Cl = 8 mL/min/kg |

| **24**, (Jin,2008)  **Gilead Sciences** | **Pyrrolloquinolone** | IC_50_ = 28 nM | IC_95_ = 7 nM  (10% FBS)  IC_95_ = 16 nM  (50% NHS) | Rat: F = 15%  T_1/2_(*i.v.*) = 1.1 h  Cl = 4.7 mL/min/kg |
| --- | --- | --- | --- | --- |
|  |  |  |  | Dog: F = 13%  T_1/2_(*i.v.*) = 0.9 h  Cl = 6.7 mL/min/kg |
| **25**, (Jin,2008)  **Gilead Sciences** | **Pyrrolloquinolone** | IC_50_ = 62 nM | IC_95_ = 7 nM  (10% FBS)  IC_95_ = 16 nM  (50% NHS) | Rat: F = 45%  T_1/2_(*i.v.*) = 4.9 h  Cl = 3.8 mL/min/kg |
|  |  |  |  | Dog: F = 16%  T_1/2_(*i.v.*) = 4.5 h  Cl = 5.7 mL/min/kg |
| **26**, (Jin,2008)  **Gilead Sciences** | **Pyrrolloquinolone** | IC_50_ = 13 nM | IC_95_ = 7 nM  (10% FBS)  IC_95_ = 16 nM  (50% NHS) | Rat: F = 4%  T_1/2_(*i.v.*) = 1.2 h  Cl = 25.5 mL/min/kg |
|  |  |  |  | Dog: F = 8%  T_1/2_(*i.v.*) = 1.8 h  Cl = 5 mL/min/kg |
